# An imported case of vaccine-derived poliovirus type 2, Spain in the context of the ongoing polio Public Health Emergency of International Concern, September 2021

**DOI:** 10.2807/1560-7917.ES.2021.26.50.2101068

**Published:** 2021-12-16

**Authors:** María Dolores Chirlaque López, María Cabrerizo, Bernardo R. Guzmán Herrador, Josefa Masa-Calles, María Ester Alarcón-Linares, Ana Allende, Esteban Aznar Cano, María Isabel Barranco Boada, Elena Cantero Gudino, Sonia Fernández-Balbuena, Ana Fernández Dueñas, María Dolores Fernández-García, Laura García Hernández, Visitación García Ortúzar, Noemí López-Perea, Eduardo Martínez-Salcedo, Antonio Moreno-Docón, María Ordobás Gavín, Inmaculada Rodero Garduño, Maria José Sierra Moros, Fernando Simón Soria, Aurora Limia Sánchez, Berta Suárez Rodríguez

**Affiliations:** 1 Department of Epidemiology, Murcia Regional Health Council, Murcia, Spain; 2 IMIB-Arrixaca, Murcia University, Murcia, Spain; 3 CIBER in Epidemiology and Public Health (CIBERESP), Madrid, Spain; 4 National Polio Laboratory, National Centre for Microbiology, Instituto de Salud Carlos III, Madrid, Spain; 5 Coordinating Centre for Health Alerts and Emergencies (CCAES), Directorate General of Public Health, Ministry of Health, Madrid, Spain; 6 National Centre of Epidemiology, Carlos III Health Institute, Madrid, Spain; 7 Research Group on Microbiology and Quality of Fruit and Vegetables, Food Science and Technology Department, CEBAS-CSIC, Espinardo, Murcia, Spain; 8 Immunization Programme Area, Directorate General of Public Health, Ministry of Health, Madrid, Spain; 9 Epidemiology and Prevention Service, Public Health General Direction, Canary Islands Health Service, Santa Cruz de Tenerife, Spain; 10 Lorca Public Health Service, Murcia, Spain; 11 Neuropediatric Unit, Department of Paediatrics, University Hospital Virgen of Arrixaca, Murcia, Spain; 12 Microbiology Service. Hospital Clínico Universitario Virgen de la Arrixaca, Murcia, Spain; 13 Sub-Directorate General of Epidemiology, Community of Madrid, Spain; 14 CIBER in Infectious Diseases (CIBERINFEC), Madrid, Spain

**Keywords:** Poliovirus, vaccine-derived poliovirus, eradication, public health emergency of international concern, Acute Flaccid Paralysis

## Abstract

The monthly retrospective search for unreported acute flaccid paralysis (AFP) cases conducted as a complementary component of the Spanish AFP surveillance system identified a case of AFP in a child admitted in Spain from Senegal during August 2021. Vaccine-derived poliovirus 2 was identified in the stool in September 2021. We present public health implications and response undertaken within the framework of the National Action Plan for Polio Eradication and the Public Health Emergency of International Concern.

In mid- to end-September 2021, a case of vaccine-derived poliovirus type 2 (VDPV2) was detected by the National Polio Laboratory in the National Centre for Microbiology (CNM) in Spain. We present the public health implications and rapid response to this event within the framework of the National Action Plan for Polio Eradication (NAPPE) [1] and the ongoing Public Health Emergency of International Concern (PHEIC) on the risk of international spread of polio [2].

## Case description

A child below the age of 6 years arrived in Murcia, south-eastern Spain, from Senegal at the beginning of August 2021. The patient was admitted to a hospital on a scheduled basis to continue supportive treatment of an acute flaccid paralysis (AFP) with unknown aetiology and with onset of symptoms in Senegal at the beginning of July 2021. In the second week of August, the patient was discharged from hospital after clinical improvement with the diagnosis of AFP secondary to acute anterior meningomyeloradiculitis because of enterovirus infection. During admission, enterovirus was found in a respiratory sample (later characterised as coxsackievirus B4) and in faeces. During the first days of September, the case stayed with a local family before returning to Senegal.

## Case detection

The NAPPE requires surveillance for early notification and virological study of all AFP cases in children under 15 years of age [3]. However, the AFP surveillance procedure was not initiated during the admission of this case. As a complementary activity of the AFP surveillance, the NAPPE states that regional public health departments (RPHD) should communicate any unreported AFP cases identified through a retrospective search in the paediatric and neurology hospitalisation wards located in their region to the National Centre of Epidemiology (CNE) on a monthly basis. Notification of zero cases is included in the system [3]. In this context, the AFP case hospitalised in August 2021, which had gone unreported, was identified by the RPHD in the Region of Murcia and notified to CNE at the beginning of September.

It was then possible to retrieve a stool sample of the case that was sent to the CNM. The presence of poliovirus (PV) was investigated by cell culture following World Health Organization (WHO) standard procedures [4,5]. A cytopathic effect was observed on the inoculated RD and L20B cells. In mid- to end-September, the virus was isolated and characterised as PV2 using the intratypic differentiation assay according to the recommended protocol [6]. Sequencing of the complete VP1 genomic region indicated that, according to the WHO-adopted criteria [7], it was a vaccine-derived poliovirus 2 (VDPV2) strain since it was 5.1% divergent from the PV2 Sabin strain in the VP1 sequence. Results were further confirmed by the Regional Reference Laboratory of the WHO/Europe for Poliomyelitis [8] at the Robert Koch Institute, Berlin, Germany.

According to the statement of the Thirtieth International Health Regulations (IHR) Emergency Committee on the international spread of PV and the ongoing PHEIC, Senegal was classified as a country with circulating VDPV2 (cVDPV2) at the time of this case’s detection in Spain. According to the WHO, the most recent detection in Senegal had been in mid-September 2021 [2]. The recent cVDPV2 outbreak detected in Senegal was linked to ongoing transmission in other areas of West Africa [9]. The case’s vaccination card showed four doses of oral polio vaccine and one dose of inactivated polio vaccine (IPV) received during the first year of life.

## National and international coordination

Following notification of the case, a national technical support group (GAT) that included all actors with a role in the management of the event was convened by the Coordinating Centre for Health Alerts and Emergencies (CCAES). Its mandate was to keep all stakeholders updated about the development of the event, discuss the information available and provide technical advice on response actions. The GAT included representatives of the CCAES and the immunisation programme area at the Ministry of Health, the CNM, the CNE and the RPHD of the Region of Murcia. A regional technical support group with all stakeholders involved in the response at the regional level was also convened in Murcia. After identifying that six contacts of the case resided in two additional regions, Madrid and the Canary Islands, representatives from RPHD of these two regions were also included in the GAT. In addition, and after proposal by the GAT, the Director General of Public Health at the Ministry of Health convened the Response Coordinating Committee (RCC) one week later (end-September). This included representatives at a higher strategic level. Actions proposed by the GAT were discussed and endorsed by the RCC.

On the same day the GAT was convened, the CCAES reported the case to the WHO Regional Office for Europe and communicated the case to the IHR focal point in Senegal, requesting additional information on the background of the case as well as on the epidemiological situation of cVDPV2. Following WHO standard operating procedures [10], the alert was classified as an event with no evidence of transmission in Spain in which VDPV2 had been detected in a human case with AFP. The case was also communicated via the Early Warning and Response System of the European Union [11].

## Measures undertaken to respond to the event

The following measures were undertaken within the framework of the NAPPE.

### Identification and classification of contacts, collection of stool samples and vaccination

Individuals who had been in contact with the case in Spain were classified into four categories according to their level of exposure, and specific actions were planned (Table 1). In Spain, there is a high immunity against polio in the general population [12,13] and since 1998, vaccination coverage at a national level has been higher than 95% [14]. In addition, coverage rates at the local level in the Region of Murcia range between 94% and 96% (data not shown). Considering this situation, and after receiving confirmation that all contacts were vaccinated according to the national immunisation programme, the RCC decided to recommend vaccination of close contacts with one dose of IPV.

**Table 1:** Classification of contacts of the case with vaccine-derived poliovirus type 2 and measures undertaken, Spain, September 2021

Classification	Definition/type of exposure	Measures	
		Collection of stool samples	Vaccination
**Close contact**			
I	Cohabitants and non-cohabitants with intense direct physical contact i.e. helped with personal hygiene, helped with feeding, played with physical contact	Collection and analysis of two faecal specimens collected > 24 h apart	One dose of IPV vaccine
II	Non-cohabitants with prolonged direct physical contact i.e. rehabilitation care		
**Casual contact**			
III	Stayed in the same room without direct physical contact	Collection of two faecal specimens collected > 24 h apart; analysis if positive samples were identified in close contacts	Considered if positive samples were identified in close contacts
IV	Any other person who has not had physical contact but has had some contact with the case i.e. family member who visited the household for a short time		

IPV: inactivated polio vaccine.

Depending on the results of the close contacts’ stool cultures, the recommendations would be reviewed in order to consider the administration of an additional dose of IPV and/or extend the vaccination recommendations. A review of potential unvaccinated individuals and possible susceptible groups was carried out in the relevant area.

Twenty close contacts and 22 casual contacts were identified, 36 in Murcia and six in other Spanish regions (Table 2). All close contacts received one dose of the IPV vaccine.

**Table 2:** Distribution of contacts of a case with vaccine-derived poliovirus type 2 by region, Spain, September 2021 (n = 42)

Region	Close contact	Casual contact	Total
Murcia	19	17	36
Madrid	0	5	5
Canary Islands	1	0	1
Total	20	22	42

### Virological analysis in contacts and in wastewater

By mid- to end-November 2021, 40 stool samples from 20 close contacts had been analysed in the CNM with the same standard cell culture methods used for the AFP case, and cVDPV2 infection was excluded for all of them. The samples of casual contacts (III and IV) are currently being studied for enterovirus only by PCR. In addition, four raw wastewater samples taken from two different entry points of the wastewater treatment plant from the area where the case had stayed on 2 different days during mid- to end-September were analysed. The samples were processed and concentrated at the CEBAS-CSIC laboratory and testing for the presence of enterovirus was carried out in the CNM by cell culture and PCR techniques. All of them were PV-negative, but non-polio enteroviruses were detected by PCR: an echovirus 3 in samples collected on the first day and an enterovirus A in samples collected on the second day.

Virological results in close contacts and wastewater samples suggest no onward PV transmission following this event in Spain.

### Enhanced surveillance with retrospective and prospective search for suspected cases

A retrospective search for potential additional cases has been conducted through a review of all admissions in all hospitals in the Region of Murcia since August 2021. A daily zero-reporting system was established to ensure prospective and timely reporting of all suspected cases from all hospitals in the Region of Murcia until the closure of the event. Two children with Guillain–Barré syndrome were identified and the AFP surveillance protocol was applied, resulting negative for PV.

### Ethical statement

Acute flaccid paralysis is a notifiable disease in Spain. No ethical approval was sought as this study describes the public health actions undertaken under the framework of the NAPPE following the detection of an imported case of AFP because of a vaccine-derived PV2 in Spain. Written consent for the case report was obtained from the legal guardian of the child.

## Discussion

The WHO European Region was declared polio-free in 2002 [15]. In light of the ongoing circulation of wild PV type 1 and cVDPV worldwide and ongoing use of live attenuated vaccines in some countries, there is a risk of introduction of wild PV, or vaccine-derived PV in countries declared polio-free such as Spain. Given the high standards of sanitation and hygiene, the high vaccination coverage and high level of immunity against PV, together with good detection and response capacities to events related to polio, the risk of transmission of PV in Spain is estimated to be low [16]. However, in the context of the NAPPE and the ongoing PHEIC, there is no room for complacency. Although indicators show that the AFP surveillance system in Spain is of good quality, its sensitivity has decreased in recent years, possibly because of a lower perception of the risk of poliomyelitis as a consequence of the absence of polio cases [17].

### Conclusion

This event has shown the importance of maintaining an optimal coordination among all the components that are part of the NAPPE and reminds us that in order to sustain a status free of PV circulation we must maintain and strengthen PV surveillance systems for early detection. This includes increasing awareness among clinicians, clinical microbiologists, public health specialists and other actors with a role in this system as well as maintaining good vaccination coverage.
